# Health-related quality of life and anxiety levels among patients under surveillance for intraductal papillary mucinous neoplasm

**DOI:** 10.1186/s12876-023-02639-0

**Published:** 2023-01-16

**Authors:** Heini Nieminen, Risto Roine, Ari Ristimäki, Eila Lantto, Norma Välimaa, Erika Kirveskari, Harri Sintonen, Caj Haglund, Hanna Seppänen

**Affiliations:** 1 Department of Surgery, Helsinki University Hospital, University of Helsinki, Haartmaninkatu 4, PL340, Helsinki, Finland; 2 Department of Health and Social Management, University of Eastern Finland, Kuopio, Finland; 3 Helsinki University Hospital, University of Helsinki, Helsinki, Finland; 4 Department of Pathology, Faculty of Medicine, Helsinki University Hospital, University of Helsinki, Helsinki, Finland; 5 HUSLAB, HUS Diagnostic Center, Helsinki University Hospital, Helsinki, Finland; 6 Department of Radiology, Paijat-Hame Central Hospital, Lahti, Finland; 7 Faculty of Medicine, University of Helsinki, Helsinki, Finland; 8 HUS Diagnostic Center, Clinical Neurophysiology, Clinical Neurosciences, Helsinki University Hospital, University of Helsinki, Helsinki, Finland; 9 Department of Public Health, University of Helsinki, Helsinki, Finland; 10 Translational Cancer Medicine, Research Programs Unit, Faculty of Medicine, University of Helsinki, Helsinki, Finland

**Keywords:** IPMN, Quality of life, Anxiety levels, IPMN surveillance, Premalignant

## Abstract

**Background:**

Because of the premalignant nature of intraductal papillary mucinous neoplasms (IPMNs), patients should undergo surveillance as long as they remain fit for surgery. This surveillance, with imaging and laboratory tests every 6 to 12 months, is expensive and may psychologically burden patients. This study aimed to determine the effects of IPMN surveillance on patients´ health-related quality of life (HRQoL) and anxiety levels.

**Methods:**

We included a random subgroup of all IPMN patients undergoing a follow-up check-up at Helsinki University Hospital (HUH) between August 2017 and November 2018. Patients were asked to complete the 15D HRQoL and state-trait anxiety inventory (STAI) questionnaires just before and three months after an IPMN control.

**Results:**

Among 899 patients in IPMN follow-up, 232 participated. The 15D HRQoL results showed differences in some IPMN patients’ 15 analyzed dimensions compared to a sex- and age-standardized general population cohort, but the clinical relevance of these differences appear doubtful. We detected no significant difference in the anxiety levels determined using the STAI questionnaires before or three months after the IPMN control.

**Conclusion:**

Surveillance should be less harmful than the risk of disease. Among our patients, the recommended IPMN follow-up carried minimal negative impact on patients’ HRQoL or anxiety levels. This result is important, because the number of patients under IPMN surveillance is rapidly increasing and the cancer risk among the majority of these patients remains small.

*Trial registration*: The Surgical Ethics Committee of Helsinki University Hospital approved this study (Dnro HUS 475/2017) and it was registered at ClinicalTrials.gov (NCT03131076) before patient enrollment began.

## Background

An intraductal papillary mucinous neoplasm (IPMN) is a cystic neoplasm of the pancreas. It can be divided into three main categories according to the location of the cyst: main duct (MD), branch duct (BD), and mixed type. As imaging for different abdominal symptoms has increased in recent years, the number of detected pancreatic cysts has also increased, thereby increasing the cumulative number of detected IPMN cysts requiring surveillance. Most IPMN findings are incidental, not causing any symptoms to the patient [1, 2]. Over time, however, some IPMNs develop increasing dysplasia, and, finally, IPMN-associated carcinoma. In a South Korean study [3] among 194 branch duct IPMN (BD-IPMN) patients, about 10% needed surgery during the first year following diagnosis. In their series, the malignancy rate reached 15% (13/194) for BD-IPMN patients during the 15-year surveillance from 1995 to 2010. Tanno et al. [1] also found that 2.5% (4/163) of IPMN patients developed a metachronous pancreatic carcinoma, a risk higher than that in the general population. BD-IPMNs generally have a good prognosis, but the minor risk of malignancy justifies and demands follow-up.

Because of the cancer risk, all IPMN patients should undergo surveillance as long as they remain fit for major pancreatic surgery. This follow-up can last for decades and includes both MRI and blood testing every 6 to 12 months as recommended in international guidelines [4, 5]. In 2015, Budde et al. published a study [6] on the socioeconomic and clinical relevance of increased IPMN detection rates, arguing that international guidelines are cost-effective and clinically relevant. Still, this follow-up protocol remains expensive and time-consuming, and as the number of IPMN patients participating continues to increase, the cumulative expenses will continue to climb as well.

Already in 1968, a World Health Organization (WHO) report by Wilson and Jungner stated that the benefits should outweigh the harm in any surveillance [7]. That is, such surveillance should not carry a larger burden than the disease itself. This criterion still appears quite relevant. Furthermore, in IPMN surveillance positive outcomes should exceed the negatives. Overbeek et al. [8] studied the psychological burden of pancreatic cyst surveillance in a cohort of pancreatic cyst patients (presumed BD-IPMN cyst in 79%; unspecified cyst in 10%). Most (94%) of the 109 patients participating reported that the advantages of surveillance outweighed the disadvantages. Moreover, one-third (33%) reported negative consequences such as discomfort and worry created by surveillance and surveillance as burdensome.

One important consideration lies in the possible negative effect of follow-up on patients’ quality of life (QoL) given an uncertain diagnosis and the fear of cancer, both of which can cause anxiety. The effects of IPMN follow-up on patients’ psychological well-being and QoL have been studied previously. For instance, Pezzilli [2] demonstrated that IPMN patients’ QoL was comparable to that in the general population and did not change during follow-up. In another Italian study comparing surgically treated IPMN patients to IPMN patients undergoing follow-up, patients being followed felt less healthy and experienced more anxiety and stress than surgical patients [9]. This result differed from a Dutch study [10], which found that high-risk individuals under pancreatic cancer surveillance only temporarily exhibited increased cancer worries and, in the long term, surveillance reduced their concern and provided a sense of certainty. Their study was conducted on patients at a higher risk for pancreatic cancer, however, not on IPMN patients. But we can assume that these two conditions are similar from the patient’s point of view.

The primary type of the IPMN tumor—main duct, branch duct, and mixed type—does not seem to impact patient QoL and psychological well-being. Pezzilli et al. [11] examined QoL, depression, and anxiety, comparing main duct and mixed-type IPMN to BD-IPMN patients. In that study, no differences were detected between the two study groups during the two-year study period. However, this study was limited by its very small number of patients (n = 9) at the end of the study.

Here, a random proportion of ambulatory IPMN patients at Helsinki University Hospital (HUH) between August 2017 and November 2018 were contacted and the effects of IPMN surveillance on patients’ health-related QoL (HRQoL) and anxiety levels were examined. We hypothesized that uncertainty and anxiety would be greatest just before the IPMN control when patients are afraid of possible malignant changes found upon imaging. We further hypothesized that HRQoL and anxiety normalizes a few months after the control if the findings remained unchanged compared to previous imaging. We conducted the study by mailing the 15D HRQoL (15D) and the state-trait anxiety inventory (STAI) questionnaires to patients before the IPMN follow-up MRI and three months after the MRI control.

## Methods

### Patients

At Helsinki University Hospital (HUH), IPMN follow-up is organized as described in international guidelines [4, 5], consisting of MRI imaging and laboratory testing every 6 to 12 months. IPMN follow-up is designed to identify possible malignant changes in IPMN cysts or through laboratory tests indicating a need for further investigations or surgery. For patients, this means surveillance continues as long as they are sufficiently fit to undergo major pancreatic surgery, which can result in decades of follow-up. Some of patients participating in IPMN surveillance at HUH were sent the 15D HRQoL and STAI questionnaires beginning in August 2017 through November 2018. All in all 899 patients were under surveillance for IPMN during that period of time and of those who received the questionnaires 232 chose to participate in the study (25,8% 232/899). The questionnaires were sent before and three months after the IPMN control MRI.

The Surgical Ethics Committee of HUH approved this study (Dnro HUS 475/2017). The study was registered at ClinicalTrials.gov (NCT03131076) before patient enrollment began. The study was carried out in accordance with relevant guidelines and regulations and informed consent was obtained from all participating patients.

### 15D health-related quality of life (15D HRQoL)

We measured patients’ HRQoL using the standardized generic 15D HRQoL questionnaire. 15D is a self-administered HRQoL measurement instrument for adults [12–14], which measures 15 different dimensions: mobility, vision, hearing, breathing, sleeping, eating, speech, excretion, usual activities, mental functioning, discomfort and symptoms, depression, distress, vitality and sexual activity. There are five levels for each dimension. 15D can be used to assess single dimensions or all dimensions together as the single index score. The single index score (15D score) represents overall HRQoL on a 0 to 1 scale (where 1 represents full health and 0 represents being dead). The dimension-level values reflecting the goodness of levels relative to no problems on a dimension (a score of 1) to being dead (a score of 0), can also be calculated from the 15D questionnaire using a set of population-based reference or utility weights. Mean dimension-level values are used to create 15D profiles for groups. The minimum clinically important change or difference in the 15D score is estimated as ± 0.015, assuming that individuals can feel such a difference on average [15]. In this study, we sought to determine if changes emerged in patients’ HRQoL before and after an IPMN control.

IPMN patients’ HRQoL results were compared to the those of an age- and sex-standardized sample from the Finnish general population collected as part of the Finnish Health 2011 Health Examination Survey [16].

### State-trait anxiety inventory (STAI)

The STAI questionnaire for adults is an instrument that measures a person’s momentary anxiety (state anxiety) and the more long-standing tendency or propensity to anxiety (trait anxiety) [17]. Both parts of the questionnaire consist of 20 questions. We used only the state anxiety portion in our study to determine patients’ anxiety levels at the time of participation. The resulting scale lies between 20 and 80 points, whereby 20 points equates with the highest possible level of anxiety and 80 points signifies the lowest level. We determined the patient STAI score before and three months after the IPMN control to determine if the IPMN control with an MRI scan impacts patients’ anxiety levels.

### Statistical analysis

Statistical analyses were performed using SPSS for Windows version 17.0 (SPSS, Inc., Chicago, IL, USA). We present results as percentages or as means (standard deviation or SD). The paired-sample t-test was used to determine the statistical significance of the differences in means between two measurement points and the independent sample t-test determined the statistical significance of the differences in means between patients and the population sample. We considered *p* < 0.05 statistically significant. Due to a possible multiple testing issue, more attention should be paid to dimensions with *p* < 0.01.

## Results

We received completed STAI and 15D questionnaires from 232 patients. The median age of participating patients was 69 years (range 29–83 years; average age, 67.6 years) and 62.9% of them were female (Table 1). During the study period, 667 patients did not participate. Table 1 also summarizes their characteristics.

**Table 1:** Characteristics of participating (n = 232) and non-participating (n = 667) IPMN patients

Patient characteristics	Participating (n = 232)	Not participating (n = 667)
Female	62.9% (n = 146)	62.2% (n = 415)
Male	37.1% (n = 86)	37.8% (n = 252)
*Age (years)*		
Min	29	30
Max	83	84
Median	69	70

The questionnaires were sent just before an IPMN follow-up. In this IPMN follow-up 20 patients of 232 had changes that needed further investigations or an earlier next follow-up. One patient was referred to pancreatic resection due to worrisome features in imaging. Two patients showed extra pancreatic tumors, one in the colon and one in the lungs.

### 15D

In the first round, we received the 15D questionnaire back from 232 patients. One patient returned an uncompleted 15D questionnaire, so that 15D profiles were available for 231 patients. The follow-up questionnaire was completed and sent to us three months later by 214 patients. The follow-up response rate was, thus, 92.2% (214/232).

**Fig. 1:**
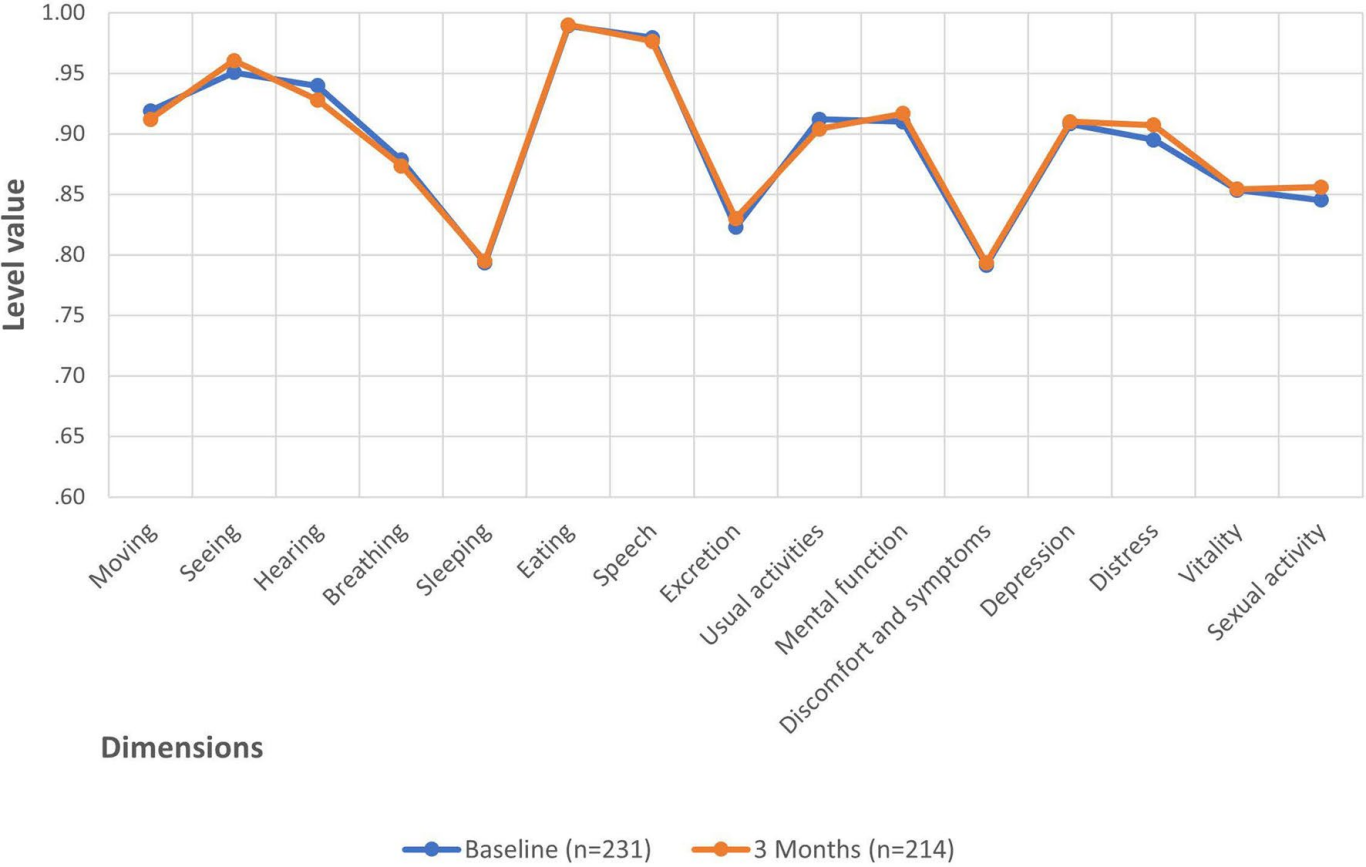
The mean 15D profiles of IPMN patients at baseline and three months later. We detected no statistically significant differences in the means between baseline (blue line, n = 231) and follow-up (orange line, n = 214). 15D score: baseline = 0.898; three months later = 0.895. Paired samples t-test: *p* = 0.339

Figure 1 reveals that only minor differences existed in the mean 15D profiles at baseline (n = 231) and at follow-up (n = 214). These differences were not statistically significant.

**Fig. 2:**
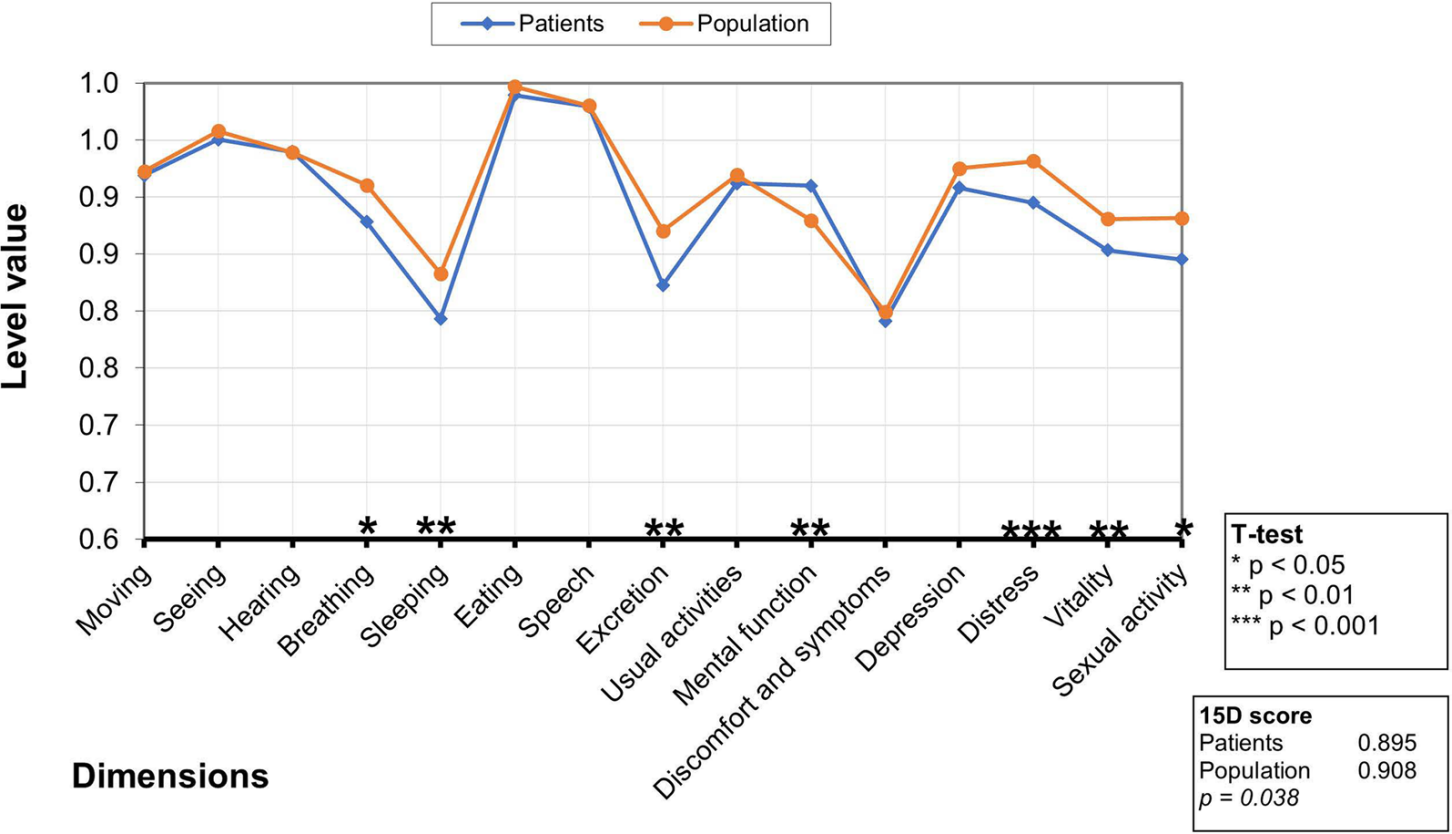
The mean 15D profile for IPMN patients at baseline (blue line) and for the age- and sex-standardized to general population (orange line). Due to a possible multiple testing issue, more attention should be paid to dimensions with more than one asterisk

The mean 15D profile for IPMN patients at baseline and for the age- and sex-standardized general population appear in Fig. 2. IPMN patients exhibited a slightly and statistically significantly higher mean score in mental functioning and slightly statistically significantly lower mean score in breathing, sleeping, excretion, distress, vitality, and sexual activity. The difference in the mean 15D score favoring the general population was statistically significant.

**Fig. 3:**
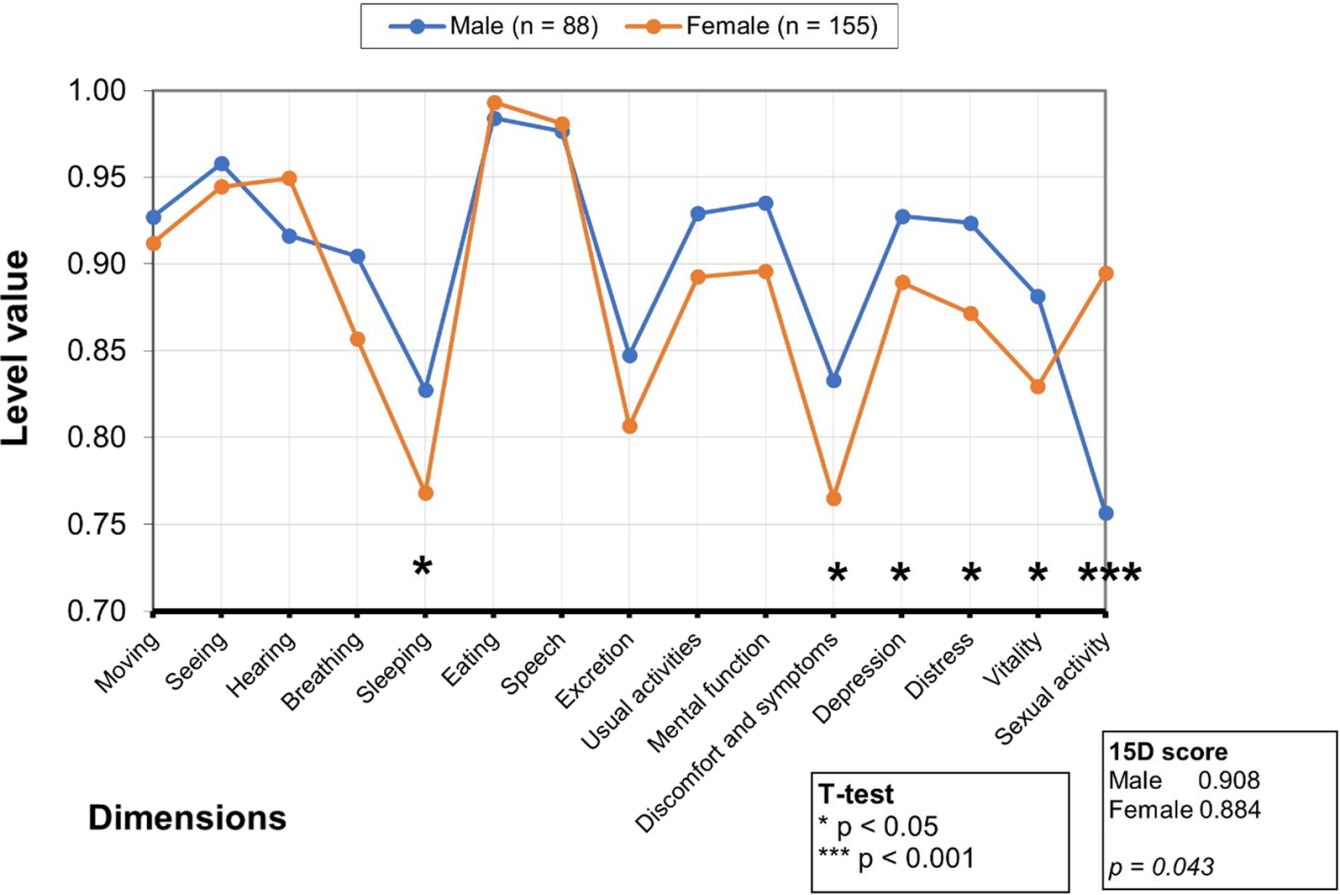
The mean 15D profiles of IPMN patients at baseline by sex. Significant differences in some of the 15D dimensions appear between male (blue line: n = 88; average age, 69) and female (orange line: n = 155; average age, 67) participants. Due to a possible multiple testing issue, more attention should be paid to dimensions with more than one asterisk

Figure 3 shows IPMN patients’ mean 15D profiles at baseline by sex. The average age was 67 among female patients and 69 among male patients. Male patients exhibited a statistically significantly lower mean score in sexual activity, while female patients had statistically significantly lower mean scores for sleeping, discomfort and symptoms, depression, distress, and vitality.

### STAI

Not all patients who returned the 15D questionnaire returned the STAI questionnaire, although the two questionnaires were sent together. In the first round, 202 of 244 patients who returned the 15D questionnaires also returned the STAI questionnaire, with 176 patients returning the STAI questionnaire in the second round. Ultimately, 159 patients completed both the baseline and follow-up STAI questionnaire allowing for a comparison of results.

**Fig. 4:**
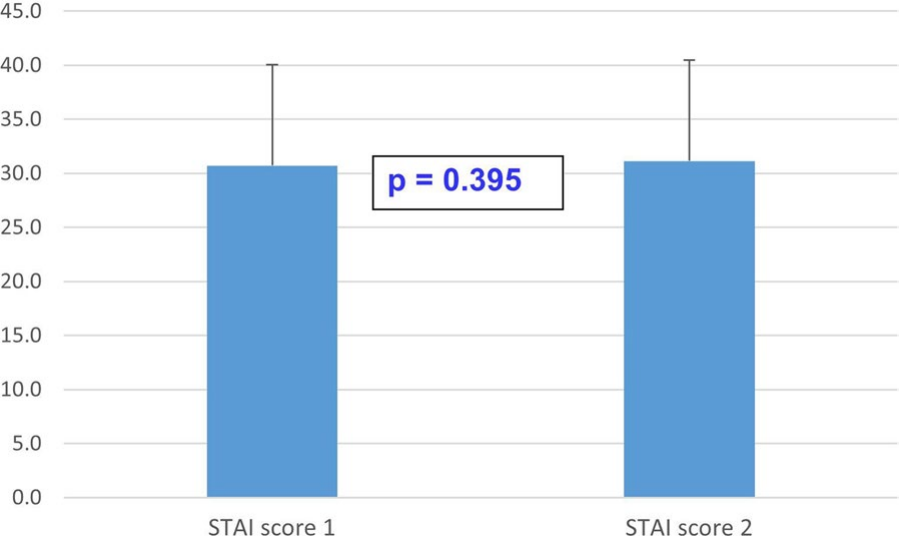
STAI scores before (STAI score 1) and three months after (STAI score 2) the IPMN control (n = 159)

Figure 4 shows the mean STAI scores of the 159 patients who responded both before and after the IPMN control. The time interval between the two measurements was on average 102 days. We detected no significant difference (*p* = 0.395) between the mean STAI scores before (mean STAI score, 30.730) and after the IPMN control (mean STAI score, 31.151).

### Age

The median age of the participating patients was 69 years. We divided the patients into two groups, younger and older than 69 years and compared these groups. There was no statistically significant difference when comparing the changes in 15D (*p* = 0.679) or STAI-results (*p* = 0.828) in these two groups.

### Length of follow-up

Of the 232 patients who returned the 15D questionnaire, 32 had been in the IPMN surveillance under 12 months, 67 patients over 12 but under 24 months, and the rest, 133 patients, over two years. The mean follow-up time was 29,7 months. The longest follow-up was over 12 years. We compared the differences in 15D score change between patients who had been in the surveillance under two years (n = 99) and over two years (n = 133) and found no statistically significant difference between the two groups (*p* = 0.347). There was also no statistically significant difference between the STAI score changes (*p* = 0.873).

## Discussion

The primary objective of this study was to investigate the possible effects of IPMN surveillance on patients’ HRQoL and anxiety levels. Our results indicate that IPMN follow-up did not negatively impact patients’ HRQoL measured using the 15D instrument. We also found no differences in the anxiety levels before and after IPMN surveillance imaging. The comparison of IPMN patients’ 15D results to those among an age- and sex-standardized population showed statistically significant differences in 7 of the 15 dimensions measured. Some statistically significant differences also emerged in the 15D dimensions between participating male and the female IPMN patients.

Age did not have an effect on the patients’ 15D- or STAI-score changes before and after the IPMN surveillance. Some studies have shown that younger patients experience higher levels of anxiety and stress when surveilled for pancreatic cancer in high-risk individuals [18, 19]. In our results we could not confirm this, but we also had only 7 patients who are under 50 years as a large proportion of the patients were older. The median age of the participants was 69.

Thirty-two of our 232 participants had been in surveillance under 12 months, 67 patients 12–23 months, and the majority, 133 patients, over 24 for months. The length of surveillance showed no statistically significant difference when comparing the patients 15D or STAI score changes. Not in IPMN surveillance, but in screening for pancreatic cancer in high-risk patient groups has been seen positive effects after one year of screening [20]. This is interesting although the study was not on IPMN patients. Also in our study there was only a three-month difference between the two measuring points.

The patient-reported effects of IPMN surveillance on HRQoL and anxiety levels were previously examined. In 2015, Pezzilli et al. [2] reported the clinical and patient-reported outcomes of 101 BD-IPMN patients. In their study, the baseline mental and physical QoL of IPMN patients did not differ from those among the general population and the results did not change during the two-year follow-up period. Our results agree with the findings that HRQoL among IPMN patients does not appear to differ from that of the general population. Furthermore, HRQoL is apparently not impacted by IPMN surveillance. Our IPMN patients reported somewhat worse results for excretion, distress, breathing, vitality, sleeping and sexual activity compared to the general population, but those differences were small and unlikely to be of clinical significance compared to the premalignant nature of IPMN tumors. It is also unclear if these differences stem from the disease itself or specifically from surveillance.

Studies similar to ours have also been performed for other diseases associated with a risk of developing malignancies. Heinonen et al. [21] studied HRQoL and anxiety in women referred for colposcopy because of an abnormal cervical cytology and human papillomavirus (HPV) infection. Like IPMN, HPV infection can be associated with cancer and requires close patient surveillance. In that study, overall HRQoL did not diminish, but the abnormal findings and referral for colposcopy were associated with diminished psychosocial components of HRQoL and anxiety. This is interesting because, like HPV infection, a rather benign disease such as BD-IPMN harbors a risk of malignancy.

We assumed that patients would be more anxious just before an IPMN control because of the worry of possible malignant changes found upon imaging. We also assumed that this worry and diminished HRQoL would normalize after the control if the patient learned that no change was detected upon imaging. Consequently, we decided to send the 15D and STAI questionnaires just before and three months after an IPMN control. During the 19 months of this study, 232 of the 899 IPMN patients under surveillance participated. We found no statistically significant difference when comparing the HRQoL and anxiety levels before and three months after IPMN follow-up. In our study, the mean anxiety levels were 30.730 (STAI score) before and 31.151 after the control, demonstrating that anxiety remained at the same high level. Yet, IPMN surveillance did not exact any negative impact on that anxiety. Furthermore, the mean 15D profiles of participating patients exhibited no statistically significant differences for any of the 15 measured dimensions before and after an IPMN control. This is comforting because the number of IPMN patients under surveillance remains high and continues to increase.

One limitation to our study lies in the patient selection. All 899 IPMN patients participating in IPMN surveillance should have received the questionnaires. But, given difficulties in mailing, this was not completely successful, and all patients did not receive the possibility to participate. Ultimately, 232 patients participated in this study. The response rate was low (25,8% 232/899), but we think that we have no reason to believe that the results would be biased. It would have been better to attempt to include all patients under surveillance in our clinic during the study period. Still, the study population we report is larger than that in other reports and, according to Table 1, no differences existed between the subgroup of patients analyzed and those not participating in this study. Therefore, we find our results reliable.

## Conclusions

Based on our results, it seems that IPMN follow-up does not impact patients’ HRQoL or anxiety levels. This is an important finding since the cohort of IPMN patients is large and continuously increasing, and follow-up typically extends across the patient’s lifetime.

## Data Availability

The datasets used and analyzed during the current study are available from the corresponding author on reasonable request.

## Abbreviations

BD: Branch duct
IPMN: Intraductal papillary mucinous neoplasm
HUH: Helsinki University Hospital
HRQoL: Health-related quality of life
MD: Main duct
MRI: Magnetic resonance imaging
STAI: State-trait anxiety inventory
PC: Pancreatic carcinoma
QoL: Quality of life
